# 

**Published:** 1911-04

**Authors:** 


					﻿UBLISHED MONTHLY.	ATLANTA	SUBSCRIPTION $1.00
JOURNAL-RECORD,
OF MEDICINE' .1
(Atlanta Medical and Surgical Journal, Established 1855. ) rr 11 y
ccessor	southern Medical Record, Established 1870^ T\Y~p>p "i j£ »
Vol. LVII.	Atlanta, Ga., April, 1911.
				

